# Risk factors for severity of thrombocytopenia in full term infants: a single center study

**DOI:** 10.1186/s13052-021-00965-1

**Published:** 2021-01-12

**Authors:** Amira M. Saber, Shereen P. Aziz, Al Zahraa E. Almasry, Ramadan A. Mahmoud

**Affiliations:** 1 Department of Pediatrics, Faculty of Medicine, Sohag University, 15 University Street, Sohag, 82524 Egypt; 2 Department of Clinical Pathology, Faculty of Medicine, Sohag University, Sohag, Egypt

**Keywords:** Neonatal thrombocytopenia, Full term infant, Rick factors, Outcome

## Abstract

**Background:**

Neonatal thrombocytopenia (NT) (platelet count < 150 × 10^9^/L) is a common finding in the neonatal intensive care unit (NICU). The main aim of this study was to assess the prevalence, risk factors, and outcomes of severe NT in full term (FT) infants.

**Methods:**

During the study period, all FT infants who met the inclusion criteria for NT on two occasions were included. Maternal data, such as maternal age, weight, gestational age, mode of delivery, and history of systemic diseases, including diabetes mellitus, pre-eclampsia, systemic lupus erythematosus, and immune thrombocytopenic purpura, were recorded. Furthermore, neonatal data, such as gender, neonatal weight, causes/duration of admission, types of respiratory support used, complete blood count measurements, and outcomes for neonates admitted to the NICU, were recorded.

**Results:**

In total, 55 FT infants with NT met the inclusion criteria, and 29 (52.73%) cases had severe NT. The most common cause of NT was neonatal sepsis (20 cases, 36.35%), followed by a postoperative state (5 cases, 9.09%). Moreover, in cases of positive blood cultures, the most commonly isolated organism was *Escherichia coli* (6 cases, 10.90%), followed by *Klebsiella* (5 cases, 9.09%). Cases of severe NT needed more platelet transfusions (*P* = 0.001) and had higher rates of mortality (*P* = 0.001) when compared to cases of mild/moderate NT associated with signs of bleeding and pulmonary/intraventricular hemorrhage (IVH) (P = 0.001).

**Conclusion:**

Severe NT compared to mild/moderate NT, associated with signs of bleeding and pulmonary/IVH, needed more platelet transfusions, and had increased mortality. Further research is needed to explain which of these complications related to severity of thrombocytopenia or were associated with original disease of the babies.

## Introduction

Thrombocytopenia, generally defined as a platelet count < 150 × 10^9^/L, affects up to 35% of all patients admitted to the neonatal intensive care unit (NICU) [1, 2]. Early onset neonatal thrombocytopenia (NT), presenting in the first 72 h of life, is commonly associated with pregnancy complications, such as intrauterine growth restriction, maternal diabetes, maternal immune thrombocytopenic purpura (ITP), congenital infection or neonatal alloimmune thrombocytopenia (NAT) [3]. While late onset NT, presenting after 72 h of life, is usually secondary to sepsis or necrotizing enterocolitis (NEC), it is usually more severe and prolonged [4].

There are two main underlying pathological mechanisms for NT: increased destruction/sequestration or decreased production of platelets. The underlying cause of NT can often be predicted by the timing of the onset of the thrombocytopenia and its natural history [5]. However, from a clinical point of view, many cases have a compound etiology for thrombocytopenia.

In most cases, NT is mild to moderate and resolves without intervention. Life-threatening bleeding, intraventricular hemorrhage (IVH), or pulmonary hemorrhage with a high risk of neurodevelopmental impairment may occur in severe NT [6, 7]. However, NT occurs less frequency in full term (FT) infants than in preterm infants, as demonstrated in one cohort study where it was 2% [8]. Moreover, the main risk factors in FT infants were occult infection, placental insufficiency, and NAT, which differs from preterm risk factors, such as sepsis, TORCH infection, and NEC [9].

Furthermore, the outcome of NT depends on several factors, such as birth weight, gestational age, platelet count and underlying cause [10]. The relationship between risk factors for NT and degree of severity in FT infants have only been researched in some studies [9, 11, 12]; however, there were no clear associations between risk factor and severity. Moreover, a clear correlation between the degree of NT and resulting bleeding risk factors has not been demonstrated [12]. Therefore, the aim of this study is to determine the risk factors and outcomes of severe NT in FT infants.

## Methods

The current clinical study was performed at the NICU in the Pediatrics Department, in cooperation with the Department of Clinical Pathology, at our University, during the period from January 2019 to the end of December 2019. Our NICU was a tertiary care level 30 beds, with 10 beds level 3, with about 1000 neonatal admission/year. Local ethical approval for the study was obtained from our Research Committee of the Faculty of Medicine (Number 652, 2018), and written informed consent was obtained from all parents of the participating children.

### Patient selection

All FT infants with a diagnosis of NT on two occasions, at the time of study, were included in the study, whether thrombocytopenia was discovered within the first complete blood count (CBC) or later. Exclusion criteria included any preterm delivery < 37 weeks gestational age or neonates who had multiple congenital malformations at birth. The severity of NT was determined according to Roberts et al. [4] and categorized into mild thrombocytopenia (platelet count 100–150 × 10^9^/L), moderate thrombocytopenia (50–99 × 10^9^/L), and severe thrombocytopenia (< 50 × 10^9^/L). Furthermore, cases were classified as early onset NT (presenting in the first 72 h of life) and late onset NT (presenting after 72 h of life). We used the following restricted guidelines for administering platelet transfusions in our unit: (I) platelet count ≤100 × 10^9^/L just going to or just having had surgery, or having clinical bleeding, (II) platelet count ≤50 × 10^9^/L and unstable (mechanical ventilation or vasopressors), and (III) platelet count of 20 × 10^9^/L and stable [6, 11].

### Maternal data collection

Fifty-five FT infants met the inclusion criteria and were enrolled in the study. Maternal data such as maternal age, weight, gestational age, mode of delivery, and maternal diseases, including diabetes mellitus, pre-eclampsia, premature rupture of membranes (PROM), systemic lupus erythematosus, ITP, and positive consanguinity, were recorded.

### Neonatal data collection and investigations

Neonatal data, such as gender, neonatal weight, APGAR score, causes of admission to NICU, duration of admission in NICU, types of respiratory support used, CBC measurements (done by Cell Dyn 3700, automated cell counter, Abbott Diagnostics, USA), and thrombocytopenic manifestations, such as purpura, ecchymosis, gastric bleeding, bleeding from puncture site, and pulmonary hemorrhage or IVH, were recorded. Detailed systemic examinations focusing on skin examination, macrosomia, head circumference, intrauterine growth retardation, congenital anomalies, and dysmorphic features were recorded. Septic work-ups and blood cultures/sensitivities were performed for all included cases. A TORCH screen was carried out (by ARCHITECT *i*1000SR, Abbott Diagnostics, USA) in cases of suspected congenital infection. Chromosomal assays [13, 14] were performed in cases of suspected chromosomal abnormities. Neonates suspected of having NAT, based on the presentation and clinic course of the illness, were designated as having an idiopathic cause as the diagnostic test for NAT is not available in our laboratory. Thrombocytopenia related morbidity were recorded in terms of pulmonary/IVH and mortality (alive or dead) for the study group.

### Statistical analysis

Data was analyzed using STATA version 14.2 (Stata Statistical Software: Release 14.2, College Station, TX: Stata Corp LP). Quantitative data was represented as mean and standard deviation, and median and range. Data was analyzed using Student’s t-test to compare the means of two groups. When the data was not normally distributed, the Mann-Whitney test was used. Qualitative data was presented as number and percentage and was compared using either the Chi square test or Fisher exact test. The *P* value was considered significant if it was < 0.05.

## Results

### Patient characteristics

In total, 55 FT infants who met the inclusion criteria were included in this study. Thirty (54.55%) cases were delivered by Caesarean section and 25 (45.45%) cases by normal vaginal delivery. Of these, 33 (60.00%) were male and 22 (40.00%) female. The mean ± SD of the platelet count at diagnosis was 67.53 ± 46.91 × 10^9^/L. In this study, 29 (52.73%) cases had severe NT at diagnosis and 26 (47.27%) cases had mild/moderate NT. Other maternal and neonatal characteristics are described in Table 1.

**Table 1:** Maternal and neonatal characteristics of studied population

Variables	Summary statistics
Mother’s age/years (mean ± SD)	29.18 ± 4.33
Mother’s weight/kg (mean ± SD)	77.36 ± 6.31
Positive consanguinity	13 (23.64%)
Mothers had Diabetes	9 (16.36%)
Mothers had Preeclampsia	6 (10.91%)
History of PROM	3 (5.45%)
Mode of delivery	
*Normal vaginal delivery*	25 (45.45%)
*Cesarean section*	30 (54.55%)
Neonatal Weight (Kg) (mean ± SD)	2.77 ± 0.61
Gender (Male/female)	33(60.00%)/ 22(40.00)
Neonatal WBCs (mean ± SD)	13.57 ± 4.83
Neonatal Hemoglobin (mean ± SD)	15.86 ± 4.46
Neonatal Platelet count at diagnosis (mean ± SD)	67.53 ± 46.91
Thrombocytopenia degree at diagnosis	
*Mild*	19 (34.55%)
*Moderate*	7 (12.73%)
*Severe*	29 (52.73%)
Manifestation of thrombocytopenia	
*No*	32 (58.18%)
*Bleeding from puncture site*	14 (25.45%)
*Gastrointestinal bleeding*	5 (9.09%)
*Pulmonary hemorrhage*	2 (3.64%)
*Intraventricular hemorrhage*	2 (3.64%)
Received Platelets transfusion	20 (36.37%)

### Causes of neonatal thrombocytopenia

As shown in Table 2, the most common cause of NT was neonatal sepsis (20 cases, 36.35%), postoperative state (5 cases, 9.09%) and idiopathic cases in (5 cases, 9.09%). Chromosomal assays were carried out for 6 patients; 2 had Down syndrome (3.70%). A TORCH screen was performed in 10 patients, and no cases had congenital infection. However, in cases of positive blood cultures, the most commonly isolated organism was *Escherichia coli* (6 cases, 10.90%), followed by *Klebsiella* (5 cases, 9.09%). Early onset NT was found in 18 (32.72%) cases, while late onset NT was found in 37 (67.27%) cases.

**Table 2:** Distribution of studied population according to some different variables

Variables	Summary (total 55 cases)
Diagnosis of thrombocytopenia	
*Late onset sepsis*	13 (23.63%)
*Early onset sepsis*	7 (12.72%)
*Post-surgery*	5 (9.09%)
*IUGR and placental insufficiency*	4 (7.27%)
*Disseminated intravascular coagulopathy*	3 (5.45%)
*Maternal ITP*	3 (5.45%)
*DM, hypoglycemia*	3 (5.45%)
*Down syndrome*	2 (3.63%)
*SLE*	2 (3.63%)
*Post exchange transfusion*	2 (3.63%)
*Tetralogy of Fallot*	2 (3.63%)
*Birth asphyxia*	2 (3.63%)
*Metabolic disorders*	2 (3.63%)
*Idiopathic*	5 (9.09%)
Onset of thrombocytopenia	
*Early onset thrombocytopenia*	18 (32.72%)
*Late onset thrombocytopenia*	37 (67.27)
Severity of thrombocytopenia	
*Mild thrombocytopenia*	19 (34.54%)
*Moderate thrombocytopenia*	7 (12.72%)
*Severe thrombocytopenia*	29 (52.73%)
Blood culture	
*No gross*	35 (63.64%)
*E-coli*	6 (10.90%)
*Klebesiella*	5 (9.09%)
*Enterobacter*	3 (5.45%)
*Pneumococci*	3 (5.45%)
*Staphylococcus aureus*	3 (5.45%)
Respiratory support	
*Room air*	15 (27.27%)
*CPAP*	25 (45.45%)
*Mechanical ventilation*	15 (27.27%)
Duration of hospital stay (mean ± SD)	8.67 ± 3.95
Outcome	
*Alive*	49 (89.10%)
*Dead*	6 (10.90%)

### Risk factors for thrombocytopenia severity

Classification of the degree of NT into mild, moderate, and severe showed that there were 19 (34.54%), 7 (12.72%) and 29 (52.73%) cases, respectively.

As shown in Table 2, 35 (63.64%) cases of NT needed respiratory support, either CPAP or mechanical ventilation, due to the presence of respiratory problems in addition to the NT. The total duration of hospital stay was 8.67 ± 3.95 days. Furthermore, in this study, 6 (10.90%) cases were declared, 5 of whom had severe NT. However, there were no significant differences in severity of NT and in maternal risk factors, such as diabetes (*P* = 0.08), pre-eclampsia (*P* = 0.09), history of PROM (*P* = 0.62), and mode of delivery (*P* = 0.51). Furthermore, in this study, the presence or absence of neonatal sepsis did not increase the severity of NT (*P* = 0.13), as shown in Table 3.

**Table 3:** Relation between thrombocytopenia severity and maternal/ neonatal characteristics

Variable	Thrombocytopenia (total 55 cases)		P value
	Mild/moderate ***N*** = 26 (47.27%)	Severe ***N*** = 29 (52.73%)	
Mother’s age/years (mean ± SD)	29.69 ± 4.77	28.72 ± 3.92	0.41
Mother’s weight/kg (mean ± SD)	77.56 ± 5.67	76.34 ± 8.23	0.62
Positive consanguinity	6 (23.08%)	7 (24.14%)	0.93
Mothers had Diabetes	5 (19.23%)	3 (10.34%)	0.08
Mothers had Preeclampsia	5 (19.23%)	1 (3.45%)	0.09
History of PROM	1 (3.85%)	2 (6.90%)	0.62
Mode of delivery			
*Normal vaginal delivery*	13 (50.00%)	12 (41.38%)	0.51
*Cesearn Section*	13 (50.00%)	17 (58.62%)	
Neonatal Weight/kg (mean ± SD)	2.77 ± 0.74	2.76 ± 0.50	0.97
Neonatal WBCs (Mean ± SD)	13.38 ± 4.45	13.74 ± 5.22	0.96
Neonatal Hemoglobin gram/dL (Mean ± SD)	15.99 ± 3.19	10.06 ± 3.46	0.001
Neonatal sepsis			
*No sepsis*	19 (73.08%)	16 (55.17%)	0.13
*Early onset sepsis*	4 (15.38%)	3 (10.34%)	
*Late onset sepsis*	3 (11.54%)	10 (34.48%)	
Manifestation of thrombocytopenia			
*No*	25 (96.15%)	7 (24.14%)	
*Bleeding from puncture site*	0	14 (48.28%)	0.001
*Gastrointestinal bleeding*	1 (3.85%)	4 (13.79%)	
*Pulmonary hemorrhage*	0	2 (6.90%)	
*Intraventricular hemorrhage*	0	2 (6.90%)	
Platelets transfusion			
*No*	26 (100%)	9 (31.03%)	0.001
*Yes*	0	20 (68.97%)	

### Thrombocytopenia manifestations

With regard to the manifestations of NT in FT infants (Table 3), most mild and moderate cases were asymptomatic (25/26 cases, 96.15%), and only one case had gastrointestinal bleeding. In contrast, 22/29 (75.86%) cases who had severe NT were symptomatic from gastrointestinal bleeding, bleeding from puncture sites, or pulmonary/IVH (*P* = 0.001). One of the idiopathic cases had severe NT and develop IVH since birth. Moreover, FT infants with severe NT needed more platelet transfusions compared to cases who had mild/moderate NT (*P* = 0.001).

### Thrombocytopenia related morbidity and mortality

As shown in Table 4, the need for invasive mechanical ventilation was associated with severe NT in 14/29 (48.28%) cases compared to only 1/26 (3.85%) cases in those who had mild/moderate NT (*P* = 0.001). Furthermore, the neonatal mortality was high in cases with severe NT (5 cases out of a total 6 cases died) compared to only one case with mild/moderate NT who died (P = 0.001).

**Table 4:** Relationship between neonatal respiratory support, duration of hospital stay, outcome and degree of thrombocytopenia

Variable	Thrombocytopenia (55cases)		P value
	Mild/moderate N = 26 (47.27%)	Severe N = 29 (52.73%)	
CPAP			
*No*	12 (46.15%)	18 (62.07%)	0.23
*Yes*	14 (53.85%)	11 (37.93%)	
Mechanical ventilation			
*No*	25 (96.15%)	15 (51.72%)	0.001
*Yes*	1 (3.85%)	14 (48.28%)	
Outcome			
*Alive*	25 (96.15%)	24 (82.76%)	0.001
*Dead*	1 (3.85%)	5 (17.24%)	
Duration of hospital stay (days)			
Mean ± SD	9.19 ± 3.70	8.21 ± 4.18	0.21
Idiopathic thrombocytopenia (N)	2/26 (7.69%)	3/29 (10.34%)	0.73

## Discussion

In this study, out of 55 FT infants who developed NT during the study period, about half of them had severe NT at diagnosis, and about two-thirds of cases appeared after 72 h of life. The most common causes of NT were neonatal sepsis and a postoperative state. Furthermore, when compared to mild/moderate NT, severe NT was associated with higher morbidity (pulmonary or IVH), needed more platelet transfusions, and had increased mortality.

In our study, severe NT was found in 52.73% of total thrombocytopenic cases. This result was higher than other studies. Gupta et al. [15] found that severe NT accounted for 34.4% of cases. In another study, 20% of cases were classified as severe NT [6]. However, in a large cohort study including 11,281 NICU admissions of term or preterm infants over 5 years, Roberts et al. [4] found that severe NT was only identified in 2.4% of cases. The reason for a higher incidence of NT in our study was probably because the incidence of sepsis in our group was high. As from the unpublished data the positive blood culture in our unit was reported in 36.6% of neonates admitted to NICU, with prevalence rate of about 6.6/1000 live births. Furthermore, the relationship between the increased severity of thrombocytopenia and rates of neonatal sepsis also shown in Charoo et al. [16]. In contrast to FT infants, in preterm babies, Christensen et al. [17] found that about 73% of extremely low birth weight infants, had at least a one-time platelet count < 150 × 10^9^/L at some time during their NICU stay, and this incidence increased up to 85% among neonates with a birth weight ≤ 800 g. Furthermore, in our study, most about two-thirds of NT cases in FT infants were late onset (after 72 h of life). This may also be due to the increased late onset sepsis in our study group. In contrast, in the Resch et al. [9] study, in which 76% of cases were born preterm, early onset NT occurred in the majority of cases (84.1%).

NT occurs more frequently in association with certain factors, such as sepsis, birth asphyxia, babies born to mothers with pre-eclampsia, and low birth weight. This was also seen in our study, where the most common cause of NT was neonatal sepsis, which occurred in about one-third of cases. Furthermore, the most commonly isolated organisms in septic neonates were gram negative (*E. coli* and *Klebsiella*) in 55% of cases. These results are in good agreement with Ree et al. [18], who found that severe NT occurred in 20% of septic neonates and the most commonly isolated organisms were gram negative. The pathogenesis of NT in neonatal sepsis is not completely understood. It has been suggested that endothelial damage activates reticuloendothelial removal of platelets in neonatal sepsis, and thrombocytopenia occurs as, ultimately, the rate of platelet production falls behind platelet consumption [2]. The second most common cause of NT in our study was a postoperative state. Although the definite causes of postoperative thrombocytopenia have not been established in the literature, many factors have been proposed, including post-transfusion dilution, infection-induced, drug-induced, heparin-induced, immune mediated, and others [19].

In NAT was an analogue of hemolytic disease of the newborn, thrombocytopenia results from transplacental passage of maternal antibodies to fetal platelets. Sensitization may occurs in the first pregnancy in about 50% of cases [4]. NAT is often associated with severe NT and may result in major bleeding, particularly IVH, which even occur intrauterine, as thrombocytopenia may occur early in pregnancy particularly in untreated women [20]. In this study, neonates suspected of having NAT were assigned as having an idiopathic thrombocytopenia as the diagnostic test for NAT is not available in our laboratory. We found no difference between NT severity and suspected NAT cases. This may be explained with the wide varieties of presentation of NAT [21].

In this study, most cases (58.18%) were asymptomatic. The most common presentations, occurring mostly with severe NT, were cutaneous bleeding from previous puncture sites and gastrointestinal bleeding. These results agree with a study by Baer et al. [6] and also agree with Park et al. [22], who found that gastrointestinal hemorrhage in patients with aplastic anemia and severe thrombocytopenia was recorded in 5% of those for whom the lowest platelet count was 20 × 10^9^/L, compared with 1% of those for whom the lowest count was 20 × 10^9^–50 × 10^9^/L.

We found that pulmonary and IVH occurred exclusively with severe NT. This agrees with studies by Setzer et al. [23], and Bolat et al. [11] as they found that lower platelet counts correlated with a higher prevalence of IVH. Until now, it was unclear whether NT caused the IVH or whether it occurred afterwards, as a result of consumptive mechanisms. In contrast to our results, Baer et al. [6] found no relationship between the lowest platelet count recorded and the presence of pulmonary hemorrhage or IVH in patients with severe NT. They speculated that factors other than NT are prominent in the pathogenesis of those varieties of neonatal bleeding, such as coagulation disorders. Duppre et al. [24] found that a cellular and humoral coagulation disorder had more of a role in the occurrence of IVH in neonates than thrombocytopenia.

In our study, there were no statistically significant differences between duration of hospital stay and severity of NT. This may be explained by the increased mortality rate in severe NT group as the babies died early in the course of illness. These results did not agree with Resch et al. [9], who found the duration of stay to be positively related to the severity of NT and the number of subsequent platelet transfusions. Furthermore, in this study, half of the neonates with severe NT required mechanical ventilation, which may explain the bad general condition of these patients, and the actual morbidity and mortality may not only be related to severe NT but also to the original disease, such as sepsis, respiratory failure, shock, postoperative state, or disseminated intravascular coagulopathy.

The outcomes of NT in our study showed that mortality increased to 10.90% with severe NT. In a study by Resch et al. [9], a mortality rate of 10.8% was significantly associated with signs of bleeding (*P* < 0.05) and correlated with an increasing number of platelet transfusions (P < 0.05), but not with the severity of NT (*P* = 0.4). Furthermore, results from studies by Baer et al. [6] and Resch et al. [9] found no relationship between the lowest platelet count recorded and the mortality rate; however, a direct relationship was observed between the number of platelet transfusions received and the mortality rate. In our study, two-thirds of cases with severe NT received at least once platelet transfusion, which may be explained by the fact that ill patients receive more platelet transfusions, or as adverse effects of platelet transfusions [25].

Our study has some limitations, mainly due to the single-center and short study time period. Nevertheless, during this one year study, 55 FT infants with NT were carefully analyzed, and cases were followed up until discharge or death; however, long term follow up may be needed in further researches. Moreover, it should be noted that diagnostic tests for NAT are not available in our lab; therefore, suspected cases were classed as idiopathic.

## Conclusion

Thrombocytopenia is a frequent challenge between neonatologists. The most common causes of NT in FT infants were neonatal sepsis, a postoperative state and placental insufficiency. Furthermore, when severe NT compared to mild/moderate NT, associated with signs of bleeding and pulmonary/IVH, required more mechanical ventilation, needed more platelet transfusions, and had increased mortality. Further research is needed to explain which of these complications related to severity of thrombocytopenia or were associated with the bad general condition of these patients due to their original disease.

## Data Availability

The datasets used and/or analyzed during the current study are available from the corresponding author on reasonable request.

## Abbreviations

CBC: Complete blood count
FT: Full term
ITP: Immune thrombocytopenic purpura
IVH: Intraventricular hemorrhage
NAT: Neonatal alloimmune thrombocytopenia
NEC: Necrotizing enterocolitis
NICU: Neonatal intensive care unit
NT: Neonatal thrombocytopenia
PROM: Premature rupture of membranes.

## References

[1] Chakravorty S, Roberts I (2012). How I manage neonatal thrombocytopenia. Br J Haematol.

[2] Sola-Visner M, Sallmon H, Brown R (2009). New insights into the mechanisms of nonimmune thrombocytopenia in neonates. Semin Perinatol.

[3] Lyori H, Fujisawa K, Akatsuka J (1997). Thrombocytopenia in neonates born to women with autoimmune thrombocytopenic purpura. Pediatr Hematol Oncol.

[4] Roberts I, Murray N (2003). Neonatal thrombocytopenia: causes and management. Arch Dis Child Fetal Neonatal Ed.

[5] Gunnink SF, Vlug R, Fijnvandraat K, Van Der Bom JG, Stanworth SJ, Lopriore E (2014). Neonatal thrombocytopenia: etiology, management and outcome. Expert Rev Hematol.

[6] Baer VL, Lambert DK, Henry E (2009). Severe thrombocytopenia in the NICU. Pediatrics..

[7] Roberts IA, Murray NA (2003). Thrombocytopenia in the newborn. Curr Opin Pediatr.

[8] Sainio S, Jarvenpaa AL, Renlund M, Riikonen S, Teramo K, Kekomaki R (2000). Thrombocytopenia in term infants: a population-based study. Obstet Gynecol.

[9] Resch E, Hinkas O, Urlesberger B, Resch B (2018). Neonatal thrombocytopenia—causes and outcomes following platelet transfusions. Eur J Pediatr.

[10] Holzhauer S, Zieger B (2011). Diagnosis and management of neonatal thrombocytopenia. Semin Fetal Neonat M.

[11] Bolat F, Kılıc SC, Oflaz MB (2012). The prevalence and outcomes of thrombocytopenia in a neonatal intensive care unit: a three-year report. Pediatr Hematol Oncol.

[12] Sola MC (2004). Evaluation and treatment of severe and prolonged thrombocytopenia in neonates. Clin Perinatol.

[13] Jordan MJ, Simons A, Schmid M. Recommendations of the international standing committee on human cytogenetic nomenclature including new sequence-based cytogenomic nomenclature. Karger; 2016.

[14] Bangs CD, Donlon TA (2005). Metaphase chromosome preparation from culturedperipheral blood cells. Curr Protoc Hum Genet.

[15] Gupta A, Mathai SS, Kanitkar M (2011). Incidence of thrombocytopenia in the neonatal intensive care unit. Med J Armed Forces India.

[16] Charoo BA, Iqbal JI, Iqbal Q, Mushtaq S, Bhat AW, Nawaz I (2009). Nosocomial sepsis-induced late onset thrombocytopenia in a neonatal tertiary care unit: a prospective study. Hematol Oncol Stem Cell Ther.

[17] Christensen R, Henry E, Wiedmeier S (2006). Thrombocytopenia among extremely low birth weight neonates: data from a multihospital healthcare system. J Perinatol.

[18] Ree IMC, Fustolo-Gunnink SF, Bekker V, Fijnvandraat KJ, Steggerda SJ, Lopriore E (2017). Thrombocytopenia in neonatal sepsis: incidence, severity and risk factors. PLoS One.

[19] Chang JC (1996). Postoperative thrombocytopenia: with etiologic, diagnostic, and therapeutic consideration. Am J Med Sci.

[20] Giovangrandi Y, Daffos F, Kaplan C, Forestier F, Mac Aleese J, Moirot M (1990). Very early intracranial haemorrhage in alloimmune fetal thrombocytopenia. Lancet..

[21] Arneth B (2020). Neonatal immune incompatibilities between newborn and mother. J Clin Med.

[22] Park YB, Lee JW, Cho BS (2010). Incidence and etiology of overt gastrointestinal bleeding in adult patients with aplastic anemia. Dig Dis Sci.

[23] Setzer ES, Webb IB, Wassenaar JW, Reeder JD, Mehta PS, Eitzman DV (1982). Platelet dysfunction and coagulopathy in intraventricular hemorrhage in the premature infant. J Pediatr.

[24] Duppre P, Sauer H, Giannopoulou EZ (2015). Cellular and hum-oral coagulation profiles and occurrence of IVH in VLBW and ELWB infants. Early Hum Dev.

[25] Dohner ML, Wiedmeier SE, Stoddard RA (2009). Very high users of platelet transfusions in the neonatal intensive care unit. Transfusion..

